# Quantitative analysis of nonadiabatic effects in dense H_3_S and PH_3_ superconductors

**DOI:** 10.1038/srep38570

**Published:** 2016-12-09

**Authors:** Artur P. Durajski

**Affiliations:** 1 Institute of Physics, Czestochowa University of Technology, Ave. Armii Krajowej 19, 42-200 Czestochowa, Poland

## Abstract

The comparison study of high pressure superconducting state of recently synthesized H_3_S and PH_3_ compounds are conducted within the framework of the strong-coupling theory. By generalization of the standard Eliashberg equations to include the lowest-order vertex correction, we have investigated the influence of the nonadiabatic effects on the Coulomb pseudopotential, electron effective mass, energy gap function and on the 2Δ(0)/*T*_*C*_ ratio. We found that, for a fixed value of critical temperature (178 K for H_3_S and 81 K for PH_3_), the nonadiabatic corrections reduce the Coulomb pseudopotential for H_3_S from 0.204 to 0.185 and for PH_3_ from 0.088 to 0.083, however, the electron effective mass and ratio 2Δ(0)/*T*_*C*_ remain unaffected. Independently of the assumed method of analysis, the thermodynamic parameters of superconducting H_3_S and PH_3_ strongly deviate from the prediction of BCS theory due to the strong-coupling and retardation effects.

The first-principles theoretical studies of the metallization and high-temperature superconductivity of dense hydrogen sulfide were reported for the first time by Li *et al*.^1^. This directly initiated the experimental work of Drozdov *et al*. who found that H_2_S compressed in a diamond anvil cell exhibits the superconductivity ranging from 30 to 150 K measured in the low-temperature runs^2^,^3^, which is consistent with calculations mentioned^1^. Furthermore, the results achieved in samples prepared at high-temperature showed that the record critical temperature of 164 K for cooper-oxide system HgBa_2_Ca_2_Cu_3_O_8+*δ*_ under quasihydrostatic pressure^4^ has been trumped. Based on a sharp drop of the resistivity to zero and an expulsion of the magnetic field, Drozdov *et al*. observed a transition from metal to superconducting state at 203 K in H_2_S sample compressed up to 155 GPa^2^,^5^. Subsequent theoretical^6^,^7^,^8^ and experimental^9^,^10^ studies suggested that at high pressure, the phase diagram favors decomposition of H_2_S into H_3_S and elemental sulfur. This result means that the superconducting state observed at 203 K comes from a decomposition product H_3_S. More recently, referring to the theoretical crystal structure searches performed by Duan *et al*.^11^, the stability of high-pressure cubic 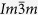 structure of H_3_S was confirmed by Li *et al*. in first-principles DFT structure searches joined with high-pressure X-ray diffraction experiments^9^, and then by Einaga *et al*. in synchrotron X-ray diffraction measurements combined with the electrical resistance measurements^10^. In contrast to cuprates where the nature of superconductivity is still not fully understood^12^,^13^,^14^, the presence of a strong isotope effect in H_3_S clearly suggests the electron–phonon origin of the superconducting state^2^,^15^,^16^,^17^. In addition, Jarlborg and Bianconi predict that the Fermi surface of H_3_S consists of multiple sheets similar to those in MgB_2_^18^. However, the existence of multi-gap superconductivity in H_3_S has not been so far confirmed.

The above theoretical and experimental discovery have stimulated significant interest in finding new hydrogen-containing superconductors^19^,^20^,^21^. Very recently, Drozdov *et al*. reported superconductivity in compressed PH_3_ with a *T*_*C*_ above 100 K^22^. The pressure dependence of the experimental critical temperature for H_3_S and PH_3_ compounds together with the relevant crystal structures is presented in Fig. 1. For H_3_S the second-order structural phase transition from trigonal *R*3*m* to cubic 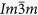 is experimentally observed at pressure close to 150 GPa^10^,^23^. However, according to the static lattice calculations the phase transition from *R*3*m* to 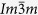 occurs at ~180 GPa^11^. A recent theoretical study found that quantum nuclear motion lowers the transition pressure to 103 GPa^24^. In the case of PH_3_, a theoretical search of crystal structure reveals two phases with lowest energy: orthorhombic *P*2_1_2_1_2_1_ and monoclinic *C*2/*m*. The DFT studies realized by Liu *et al*. indicate that both structures are dynamically stable and superconducting but *C*2/*m* phase are in a better agreement with an experimental results^25^. Unfortunately, the lack of structural informations on the superconducting phases from suitable measurements do not allow at this moment for the unambiguous verification of these assumptions.

Motivated by a recent significant experimental and theoretical progress in chemistry and physics of hydrogen-dense materials, we have carried out calculations to explore in detail the thermodynamic properties of superconducting hydrogen sulfide H_3_S and hydrogen phosphide PH_3_ at extremely high pressure (*p* = 200 GPa). The very large values of electron-phonon coupling interaction observed in these systems, caused that our investigations were performed within the framework of the Migdal-Eliashberg (ME) theory of superconductivity^26^, which goes beyond the BCS model^27^ by taking into account the retardation and strong-coupling effects.

This paper is organized as follows. In Section II, we introduce the theoretical model used to determine the quantities characterizing the superconducting state. Moreover, we present details of the first-principles calculations carried out to study the phonon properties and electron-phonon interactions. Then, in Section III, we report and compare the thermodynamic properties of superconducting H_3_S and PH_3_ systems at 200 GPa. We discuss the validity of the conventional Migdal-Eliashberg theory by introduce the lowest-order vertex correction and we examine its effect on Coulomb pseudopotential, energy gap, 2Δ(0)/*T*_*C*_ ratio and electron effective mass. Finally, we give a summary of this study in Section IV.

## Theoretical model and computational methods

Besides the experimental works, most of the theoretical studies concluded that compressed H_3_S and PH_3_ are phonon-mediated strong-coupling superconductors^8^,^11^,^25^,^28^,^29^. Thus, the superconducting state of these compounds can be accurately described by the Migdal-Eliashberg theory^30^,^31^. The Eliashberg equations for the superconducting order parameter function *φ*_*n*_ ≡ *φ(iω*_*n*_) and the electron mass renormalization function *Z*_*n*_ ≡ *Z(iω*_*n*_) written in the imaginary-axis formulation are given by^26^,^32^:


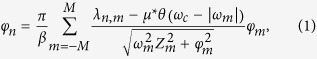


and


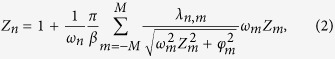


where the pairing kernel for the electron-phonon interaction is given by:


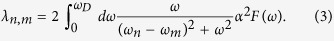


Moreover, *β* = 1/*k*_*B*_*T* and *k*_*B*_ = 0.0862 meV/K states the Boltzmann constant. Symbols μ* and *θ* denote the screened Coulomb repulsion and the Heaviside function with cut-off frequency *ω*_*c*_ equal to ten times the maximum phonon frequency: *ω*_*c*_ = 10*ω*_*D*_.

The application of the above Eliashberg equations to describe the electron-phonon superconductivity is justified for systems in which the value of the phonon energy scale (Debye frequency, *ω*_*D*_) to the electron energy scale (Fermi energy, *ε*_*F*_) ratio is negligible. Otherwise the Eliashberg equations should be generalized by taking into account the lowest-order vertex correction^33^,^34^,^35^,^36^,^37^:


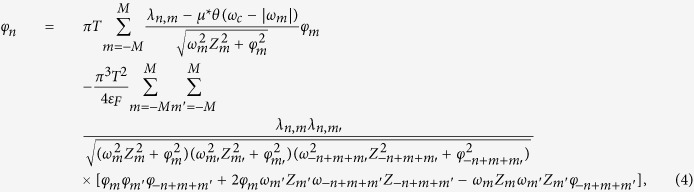


and


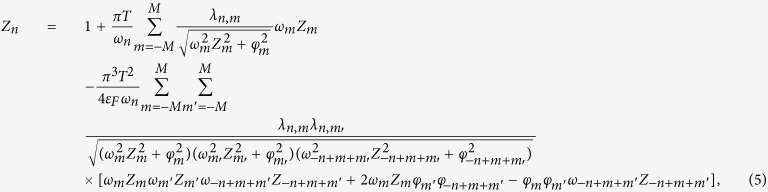


where, the modified electron-phonon pairing kernel takes the following form:


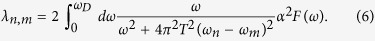


**Figure 2:**
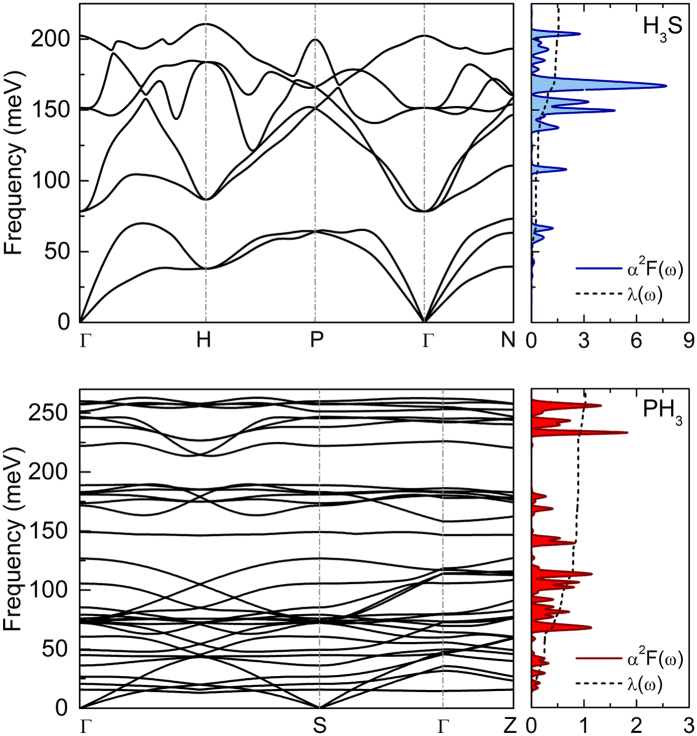
Phonon dispersion and the Eliashberg spectral function *α*^2^*F(ω*) with electron-phonon integral *λ*(ω) for H_3_S and PH_3_ superconductors at 200 GPa.

The Eliashberg spectral function, one of the main input element to the Eliashberg equations, is defined as:


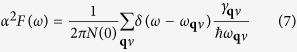


with


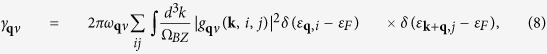


where *N*(0), *γ*_**q***ν*_ and *g*_**q***ν*_(**k**, *i, j*) denote the density of states at the Fermi energy, the phonon linewidth and the electron-phonon coefficients, respectively. The *α*^2^*F(ω*) functions for H_3_S and PH_3_ were calculated in this paper using density functional perturbation theory and the plane-wave pseudopotential method, as implemented in the Quantum-Espresso package^38^. We assume that H_3_S has cubic crystal 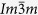 structure with lattice parameter *a* = 2.984 Å^11^, whereas PH_3_ crystallize in monoclinic *C*2/*m* structure with lattice parameter *a* = 5.152 Å, *b* = 2.961 Å, *c* = 2.960 Å, *α* = *γ* = 90° and *β* = 90.23° 39. The Vanderbilt-type ultrasoft pseudopotentials for S, P and H atoms were employed with a kinetic energy cutoff equal to 80 Ry. The phonon calculations were performed for 32 × 32 × 32 Monkhorst-Pack *k*-mesh with gaussian smearing of 0.03 Ry. The electron-phonon coupling matrices were computed using 8 × 8 × 8 *q*-grid for H_3_S and 4 × 4 × 4 *q*-grid for PH_3_. The calculated phonon band structures together with the Eliashberg spectral functions and electron-phonon integrals 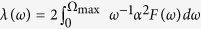 for both investigated hydrides are presented in Fig. 2. The absence of imaginary frequencies in the full phonon spectra indicates that both systems are dynamically stable.

**Figure 3:**
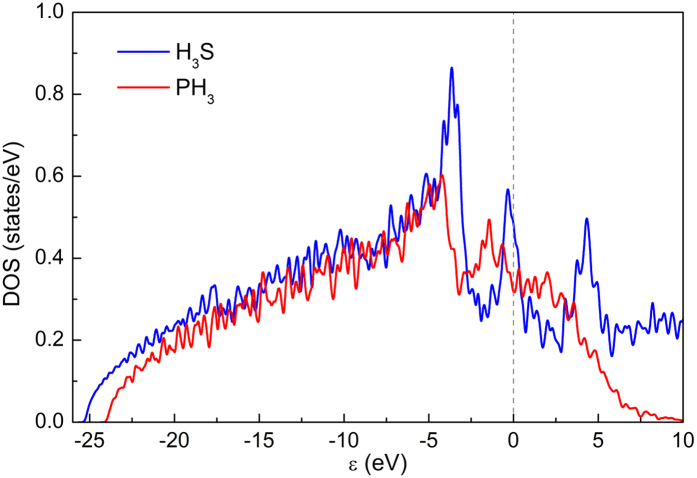
Density of states (DOS) for H_3_S (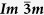) and PH_3_ (*C*2/*m*) at 200 GPa. The dotted line at zero indicates the Fermi level.

The electronic band structures of H_3_S and PH_3_ at 200 GPa were also explored. The investigated compounds are good metals with a large density of states (DOS) at the Fermi level. To clarify the difference of the electronic structures between 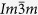 H_3_S and *C*2/*m* PH_3_, the DOS calculated for these two crystal structures are shown in Fig. 3. A strong peak around the Fermi level (the Van Hove singularities) is favorable for a strong electron-phonon coupling and thus really high superconducting temperature in the case of H_3_S^40^.

Our *ab-initio* studies showed that ratio *λω*_*D*_/*ε*_*F*_ is equal to 0.020 for H_3_S and 0.014 for PH_3_. These values are rather small in comparison to fullerene compounds or high-*T*_*c*_ cuprates, however, are not zero. Due to the above, we decided to conduct our calculations simultaneously using the conventional Eliashberg equations and equations with the lowest-order vertex correction, which allows us to examine the influence of nonadiabatic effects on the thermodynamic properties in studied compounds. The numerical analysis was performed using a self-consistent iteration methods^41^, which were implemented successfully in our previous papers^31^,^42^,^43^,^44^. The convergence and precision of our results are controlled by assuming the sufficiently high number (*M* = 1100) of Matsubara frequencies *ω*_*n*_ ≡ (*π/β*)(2*n* − 1), where *n* = 0, ±1, ±2, …, ±*M*.

## Results and Discussion

**Figure 4:**
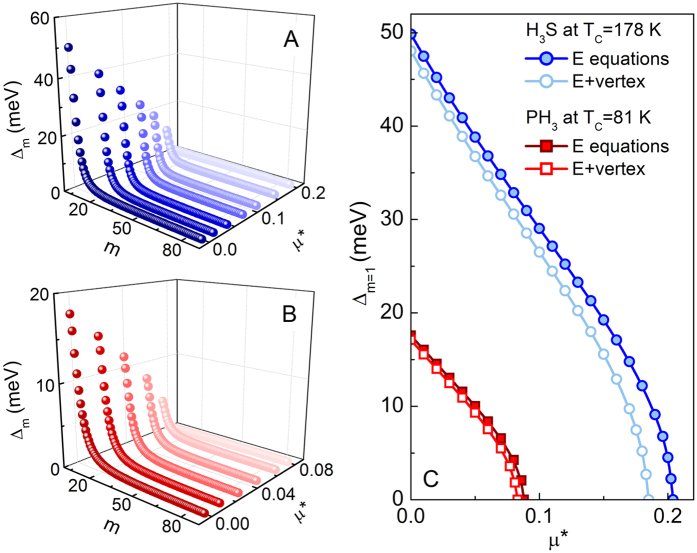
The order parameter on the imaginary axis as a function of *m* and μ* for (**A**) H_3_S and (**B**) PH_3_ at 200 GPa - the results was obtained using conventional Eliashberg (E) equations. (**C**) The full dependence of the first value of the order parameter as a function of Coulomb pseudopotential.

To study the thermodynamic properties of phonon-mediated superconductors on the quantitatively level in the first step we determine the critical value of the Coulomb pseudopotential 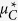. For this purpose, in the Eliashberg equations we replace *T* by the experimental value of critical temperature: *T*_*C*_ = 178 K for H_3_S^2^ and *T*_*C*_ = 81 K for PH_3_^22^. Then, starting from zero, we increase the value of μ* until we reach the equality 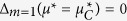, where the order parameter is defined as: Δ_*m*_ = *φ*_*m*_/*Z*_*m*_^45^. The obtained results are presented in Fig. 4. In particular, on the basis of the full dependence of Δ_*m*=1_(μ*) we can conclude that if we take into account the conventional Eliashberg equations, the Coulomb pseudopotential takes a relatively high critical value for H_3_S (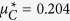) and low value for PH_3_ (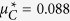) at 200 GPa. Moreover, at this point we can found that, for a fixed value of critical temperature, the lowest-order vertex correction changes 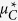 by −9.3% for H_3_S and −5.7% for PH_3_.

**Figure 5:**
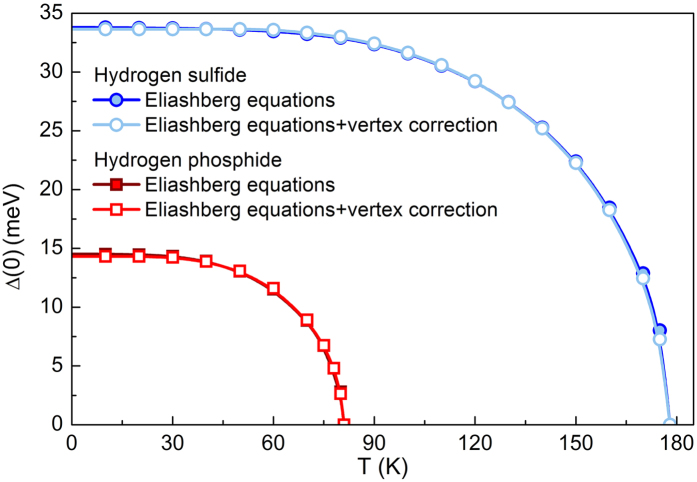
The temperature dependence of the superconducting energy gap. The numerical results can be reproduced using the analytical formula 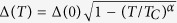, where *α* = 3.36.

In the next step, by using the analytical continuation of the imaginary-axis solutions to the real frequency axis^46^, we calculate the temperature dependence of superconducting energy gap Δ(*T*) = Re[Δ(*ω* = Δ(*T*), *T*)]^45^. The obtained results are presented in Fig. 5. Knowledge of the energy gap value at zero temperature allowed us to calculate the dimensionless ratio *R*_Δ_ ≡ 2Δ(0)/*T*_*C*_ for which the BCS theory predicts universal value [*R*_Δ_]_BCS_ = 3.53. In the case of studied hydrides, *R*_Δ_ exceed the value of 4, in particular by using the conventional Eliashberg equations we received 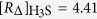 and 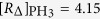. This behavior is connected with the strong-coupling and retardation effects, which in the framework of the Eliashberg formalism can be characterized by the ratio *T*_*C*_/*ω*_ln_, where *ω*_ln_ is the logarithmic phonon frequency, correspondingly 131 meV for H_3_S and 79 meV for PH_3_. Thus, the considered ratio equals 0.12 and 0.09, respectively, while in the weak-coupling BCS limit we have: *T*_*C*_/*ω*_ln_ → 0. Moreover, we find that although the vertex corrections seriously reduce 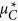 for H_3_S and PH_3_, the ratio 2Δ(0)/*T*_*C*_ remains practically unaffected. Similar situation is observed in the case of electron effective mass at *T*_*C*_ calculated from 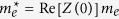, where *m*_*e*_ denotes the electron band mass. In our case 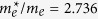 for H_3_S and 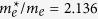 for PH_3_, regardless of the equations applied. This means that the thermodynamic properties of phonon-mediated superconductors can be successfully obtained in the framework of the conventional Migdal-Eliashberg formalism with the proviso that 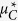 has to be accurately determined.

In the last step, to investigate the specific heat and thermodynamic critical field behavior, the condensation energy was numerically calculated:


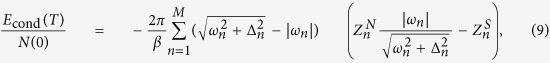


where 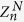 and 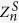 denote the mass renormalization functions for the normal and superconducting states, respectively. The specific heat difference between superconducting and normal state was then obtained from the second derivative of *E*_cond_(*T*):


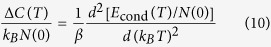


and thermodynamic critical field is defined as:


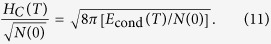


**Figure 6:**
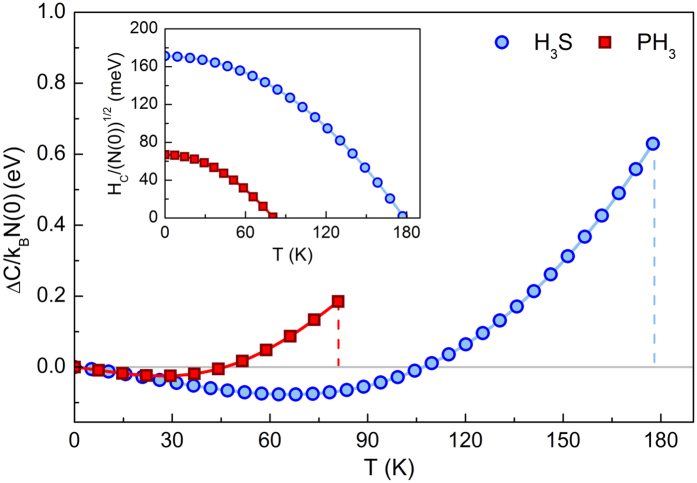
The specific heat difference between the superconducting and the normal state as a function of temperature. Inset presents the temperature dependence of the thermodynamic critical field.

The temperature dependence of Δ*C*, with characteristic specific heat jump at *T*_*C*_ marked by vertical line, is presented in Fig. 6. The inset shows the thermodynamic critical field for investigated hydrides. These results allow us to determine the other two fundamental dimensionless ratios: 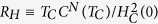 and *R*_*C*_ ≡ Δ*C(T*_*C*_)/*C*^*N*^(*T*_*C*_), where the specific heat for the normal state is defined as *C*^*N*^ = *γT*, and *γ* denotes the Sommerfeld constant: 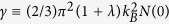. It is noteworthy that, in the framework of the BCS theory, these ratios adopt universal values of 0.168 and 1.43, respectively^45^. We emphasize that these dimensionless ratios, similar to that of energy gap, take non-BCS values, in particular: *R*_*H*_ = 0.136, *R*_*C*_ = 2.47 for H_3_S and *R*_*H*_ = 0.150, *R*_*C*_ = 1.99 for PH_3_. We can see that with increasing values of *T*_*C*_/*ω*_*ln*_ ratio the thermodynamic properties take increasingly non-BCS behavior.

## Conclusions

In this work, using the first-principles calculations and Eliashberg theory with and without vertex corrections, we systematically study the nonadiabatic effects on the superconductivity of compressed H_3_S and PH_3_ compounds. We find that for a fixed experimental value of critical temperature the lowest-order vertex correction reduces the Coulomb pseudopotential (from 0.204 to 0.185 in the case of H_3_S and from 0.088 to 0.083 for PH_3_), however, the energy gap, electron effective mass and ratio 2Δ(0)/*T*_*C*_ remain unaffected. It means that the superconducting behavior can be properly determined even in the framework of the classical Migdal-Eliashberg formalism, as long as the value of the Coulomb pseudopotential is correctly determined. Moreover, we calculated the specific heat and thermodynamic critical field and we proved that strong-coupling and retardation effects caused that thermodynamic properties of H_3_S and PH_3_ at high pressures cannot be correctly estimated in the framework of the BCS theory.

## Additional Information

**How to cite this article**: Durajski, A. P. Quantitative analysis of nonadiabatic effects in dense H_3_S and PH_3_ superconductors. *Sci. Rep.* **6**, 38570; doi: 10.1038/srep38570 (2016).

**Publisher's note:** Springer Nature remains neutral with regard to jurisdictional claims in published maps and institutional affiliations.

## Figures and Tables

**Figure 1:**
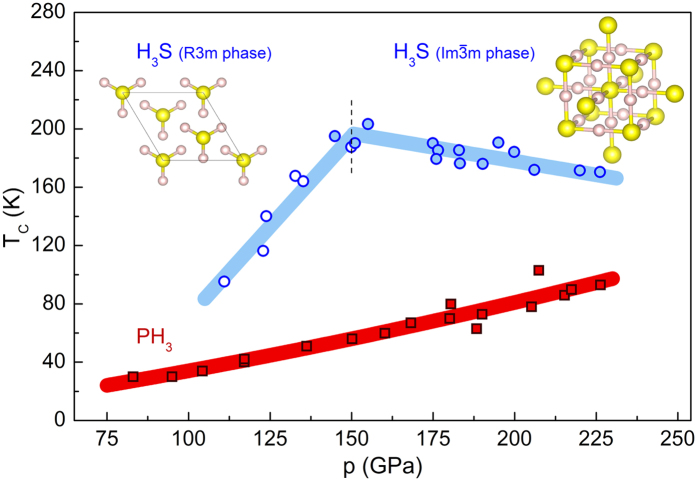
The experimental data of critical temperature as a function of pressure for H_3_S^2^,^10^ and PH_3_^22^. In addition the stable structures of H_3_S^11^ are presented.
